# Evidence of Cooperation between Hippo Pathway and *RAS* Mutation in Thyroid Carcinomas

**DOI:** 10.3390/cancers13102306

**Published:** 2021-05-12

**Authors:** Thaise Nayane Ribeiro Carneiro, Larissa Valdemarin Bim, Vanessa Candiotti Buzatto, Vanessa Galdeno, Paula Fontes Asprino, Eunjung Alice Lee, Pedro Alexandre Favoretto Galante, Janete Maria Cerutti

**Affiliations:** 1 Genetic Bases of Thyroid Tumors Laboratory, Division of Genetics, Department of Morphology and Genetics, Escola Paulista de Medicina, Universidade Federal de São Paulo, Pedro de Toledo 669, 11 Andar, São Paulo, SP 04039-032, Brazil; thaise.nayane@unifesp.br (T.N.R.C.); larissa.bim@unifesp.br (L.V.B.); 2 Centro de Oncologia Molecular, Hospital Sírio-libanês, Rua Professor Daher Cutait 69, Bela Vista, São Paulo, SP 01308-060, Brazil; vbuzatto@mochsl.org.br (V.C.B.); vgaldeno@mochsl.org.br (V.G.); pasprino@mochsl.org.br (P.F.A.); pgalante@mochsl.org.br (P.A.F.G.); 3 Division of Genetics and Genomics, Boston Children’s Hospital and Harvard Medical School, 3 Blackfan Circle, CLS (Center for Life Science) Building 15th Floor, Office 15020 | Lab 15072, Boston, MA 02115, USA; ealice.lee@childrens.harvard.edu

**Keywords:** thyroid cancer, *RAS* mutation, RNA-Seq, TCGA, Hippo pathway

## Abstract

**Simple Summary:**

*RAS* mutations have been reported in a wide range of thyroid cancer histological types, from benign to aggressive phenotypes. The presence of *RAS* mutations in benign lesions suggests that the mutation alone is unlikely to lead to a malignant transformation per se, and thus, additional aberrations are necessary for this transformation. In this study, we initially screened a cohort of 120 thyroid carcinomas with a panel of known driver mutations and identified 11 *RAS*-mutated samples. An RNA-Seq analysis of those 11 *RAS*-positive samples identified that the Hippo pathway was both mutated and differentially expressed in the *RAS*-positive tumors. The gene expression analysis of 60 *RAS*-positive The Cancer Genome Atlas (TCGA) papillary thyroid carcinomas (PTC) samples supported our findings.

**Abstract:**

Thyroid cancer incidences have been steadily increasing worldwide and are projected to become the fourth leading cancer diagnosis by 2030. Improved diagnosis and prognosis predictions for this type of cancer depend on understanding its genetic bases and disease biology. *RAS* mutations have been found in a wide range of thyroid tumors, from benign to aggressive thyroid carcinomas. Based on that and in vivo studies, it has been suggested that *RAS* cooperates with other driver mutations to induce tumorigenesis. This study aims to identify genetic alterations or pathways that cooperate with the *RAS* mutation in the pathogenesis of thyroid cancer. From a cohort of 120 thyroid carcinomas, 11 *RAS*-mutated samples were identified. The samples were subjected to RNA-Sequencing analyses. The mutation analysis in our eleven *RAS*-positive cases uncovered that four genes that belong to the Hippo pathway were mutated. The gene expression analysis revealed that this pathway was dysregulated in the *RAS*-positive samples. We additionally explored the mutational status and expression profiling of 60 *RAS*-positive papillary thyroid carcinomas (PTC) from The Cancer Genome Atlas (TCGA) cohort. Altogether, the mutational landscape and pathway enrichment analysis (gene set enrichment analysis (GSEA) and Kyoto Encyclopedia of Genes and Genome (KEGG)) detected the Hippo pathway as dysregulated in *RAS*-positive thyroid carcinomas. Finally, we suggest a crosstalk between the Hippo and other signaling pathways, such as Wnt and BMP.

## 1. Introduction

The incidence of thyroid cancer has been steadily increasing worldwide in both men (5.4%) and women (6.5%), and it is projected to become the fourth leading cancer diagnosis by 2030 [1]. Hence, it is essential to prepare for the increasing rate of diagnosis and to better discriminate the low-risk disease that may represent the early stage of the high-risk disease from those low-risk diseases that are indolent, and active surveillance has been proposed as the treatment modality. It is also important to predict the high-risk disease that will almost surely do poorly and improve the treatment of patients with advanced and aggressive disease. Ongoing efforts to improve the diagnosis and better predict the prognosis for thyroid cancer depend on a deep and more precise understanding of the genetic bases and disease biology of this tumor. 

The Cancer Genome Atlas (TCGA), a landmark cancer genomics program, sequenced the genomes and transcriptomes of nearly 500 papillary thyroid carcinomas (PTC), the most common type of thyroid cancer, to identify novel drivers (e.g., genes or mutations) that could improve the molecular diagnosis of thyroid cancer [2]. They also developed a 71-gene expression signature, named the BRAF-RAS Score (BRS), designed to quantify the gene expression profile of a given tumor that resembles either the BRAF V600E or *RAS*-mutant profiles, the most common mutations found in PTC. Using this approach, PTC samples were, therefore, divided into *BRAF*-like or *RAS*-like groups [2]. 

Although it has been created a comprehensive atlas of the somatic genetic changes involved in the pathogenesis of classical variant of PTC cases, that is not completely true for other thyroid carcinomas, such as the follicular variant of papillary thyroid carcinomas (FVPTC), follicular thyroid carcinomas (FTC) and Hurthle cell carcinoma (HCC) [3,4,5,6,7,8,9]. Additionally, while the BRAF-like group has been widely investigated, studies that have analyzed molecular differences in the *RAS*-like group are limited. 

Importantly, *RAS* mutations have been found in a wide range of thyroid tumors, from benign follicular thyroid adenoma (FTA) and noninvasive follicular thyroid neoplasm with papillary-like nuclear features (NIFTP), to poorly differentiated thyroid carcinomas (PDTC) and undifferentiated thyroid carcinomas (UTC) [10,11,12,13,14,15,16,17].

Although mutations in the *RAS* gene predispose cells to large-scale genomic abnormalities [18], it has been suggested that *RAS* induces growth arrest and premature senescence in primary cells, unless it cooperates with other driver mutations [19]. Some in vivo studies confirmed that the *RAS* mutation alone is not sufficient to trigger a thyroid transformation and is unable to completely activate the MAPK pathway [20,21,22,23,24,25]. However, double-mutant mice, *KrasG12D* and Pten−/−, developed invasive and metastatic FTC [24].

Genomic studies on PDTCs and UTCs have unveiled that mutations in *RAS* genes co-occur with mutations in other genes, such as *TERT*, *TP53, EIF1AX* or *PTEN*, as well as with the 22q loss of heterozygosity [10,26,27,28]. It has also been shown that the acquisition of an activating *KRAS* G12V mutation acts as a potential resistance mechanism in patients with BRAF V600E-mutated PTC and treated with BRAF and MEK inhibitors [29].

Altogether, these findings suggest that *RAS* mutations do not lead to a malignant transformation per se; thus, additional mutations are necessary for this transformation. In this study, we aimed to identify the genetic alterations or pathways that cooperate with *RAS* mutations in the pathogenesis of thyroid cancer. We screened a cohort of 120 thyroid carcinomas and identified 11 *RAS*-mutated samples that were subjected to RNA-Sequencing (RNA-Seq) analyses. We further evaluated 60 *RAS*-positive PTCs from the TCGA cohort. We identified mutations in novel genes and a pathway that likely cooperates with *RAS* in thyroid tumor pathogenesis.

## 2. Materials and Methods

### 2.1. Patients 

This study was approved by the institutional Research Ethics Committee of the Universidade Federal de São Paulo (UNIFESP) and Universidade de São Paulo (USP). The study included patients who underwent thyroidectomy at Hospital São Paulo (Universidade Federal de São Paulo) and Hospital das Clínicas (Faculdade de Medicina, Universidade de São Paulo) as the initial treatment for thyroid cancer and received no prior treatment for their disease. A portion of the thyroid specimen was taken from the patient at the time of surgery, snap-frozen and stored at −80 °C. The original cohort was comprised of 120 fresh-frozen samples obtained from patients diagnosed with thyroid cancer. The validation cohort included an independent set of formalin-fixed, paraffin-embedded (FFPE) surgical specimens that were obtained from the files of the Department of Pathology from UNIFESP. This set of samples included five *RAS*-positive and five BRAF V600E-positive samples, as well as five normal adjacent thyroid tissues. H&E-stained (Hematoxylin and Eosin) tissue sections from each tumor were reviewed by a pathologist to confirm the diagnosis.

### 2.2. Study Design 

**Figure 1:**
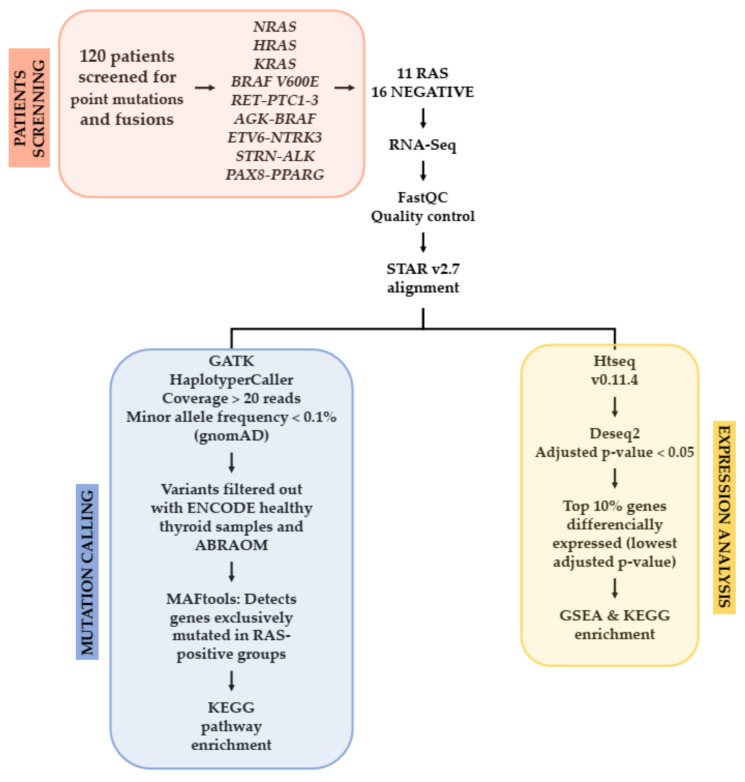
Workflow of the sample selection, screening and RNA-Sequencing pipeline analysis for the discovery cohort.

A total of 120 specimens from patients with PTC, FVPTC and FTC were screened for a panel of point mutations (*K-N-HRAS* and *BRAF V600E*) and fusions (*RET-PTC1, RET/PTC2, RET/PTC3, AGK-BRAF, ETV6-NTRK3, STRN-ALK* and *PAX8-PPARG*). The discovery cohort was comprised of eleven samples (5 FTC and 6 FVPTC) confirmed as positive for *RAS* mutations. The control group included sixteen samples negative for the panel of mutations and fusions. Twenty-seven samples were submitted to RNA-sequencing in an Illumina NextSeq platform (Figure 1). 

The demographic; clinical and pathological features, such as age at diagnosis, gender, histological subtype, tumor size, tumor focality, extrathyroidal extension, vascular invasion, presence of metastasis and *RAS* mutational profile, are detailed in Table 1.

### 2.3. RNA and DNA Isolation 

For the RNA-Seq experiment, total RNA was isolated from fresh-frozen samples using TRIzol reagent (Invitrogen Corp., Carlsbad, CA, USA) according to the manufacturer’s instructions. RNA Integrity Number (RIN) was measured using an Agilent 2100 Bio-analyzer (Agilent Technologies Inc., Santa Clara, CA, USA). To validate our findings, FFPE thyroid tissue sections were used. Representative tumor blocks containing a percentage of tumor cells greater than 70% were preferentially selected. For samples containing less than 70% of tumor cells, the tumor-containing area was marked by the pathologist and manually macro-dissected to enrich the proportion of neoplastic cells. DNA and RNA were isolated from FFPE sections using the NucleoSpin Tissue Kit (Macherey-Nagel GmbH & Co. KG, Düren, Germany) and RNeasy FFPE Kit (Qiagen, Hilden, Germany), respectively. Nucleic acid was quantified using a NanoDrop spectrophotometer (Thermo Fisher Scientific Inc., Waltham, MA, USA).

### 2.4. RNA Library Preparation 

Briefly, 0.5–1 micrograms of RNA from each sample were used to prepare the sequencing libraries. RNA libraries were prepared using a TruSeq Total RNA Sample preparation kit v.2 (Illumina Inc., San Diego, CA, USA), according to the manufacturer’s protocols. Library concentrations and quality were measured using a Qubit fluorometer and Quant-iT RNA kit (Life Technologies, Carlsbad, CA, USA). Samples were sequenced for paired-end reads on an Illumina NextSeq 500 platform (Illumina Inc.), targeting 100 million raw reads per sample, at the Centro de Oncologia Molecular, Hospital Sírio-libanês, São Paulo, Brazil.

### 2.5. RNA-Sequencing and Data Analysis

RNA-Seq reads were assessed for quality control using the FastQC format (Babraham Institute, Babraham, UK). The total set of paired-end reads was mapped against the human genome (GRCh38) for gene expression analysis and mutation calling (GRCh37) using STAR (version 2.7; default parameters with transcript-aware) [30]. Transcript abundance was estimated using HTSeq count (version 0.11.4; parameters -m union -s reverse -a 20) [31] and GENCODE (version 24) as reference for the human transcriptome. Nucleotide variants (Single-Nucleotide Variations (SNVs) and Insertions or Deletions (InDels)) were called using Genomic Analysis Toolkit (GATK) HaplotyperCaller [32]. We excluded the reads with low mapping quality (Q < 20) and further selected only variants with minor allele frequencies (MAF) reported by a genome aggregation database (gnomAD) lower than 0.1%. We used healthy thyroid samples from the ENCODE database (https://www.encodeproject.org, accessed on 20 February 2019) as the normal control. 

The mutation-calling pipeline was the same described for tumor samples, and the variants found in normal thyroid tissue were filtered out from tumor mutation results. We subsequently recalled only variants with predicted damaging impact scores on the protein function produced by SIFT and PolyPhen-2, which are inserted in the dbNSFP database [33]. Finally, we selected the variants likely pathogenic (by SIFT and PolyPhen-2) and that were not represented at the Brazilian genomic variants (ABraOM) database [34]. Maftools [35] was used to detect genes exclusively mutated in *RAS* samples (default parameters: min mut = 2, *p*-value ≤ 0.1, odds ratio analysis) compared to 16-gene mutation panel negative samples. Genes found exclusively mutated were enriched using the Kyoto Encyclopedia of Genes and Genome (KEGG) database through Enrichr (https://maayanlab.cloud/Enrichr, accessed on 28 July 2020). The workflow with the pipeline to the RNA call variants and expression is detailed in Figure 1.

### 2.6. Experimental Validation by Sanger Sequencing

We carried out Sanger sequencing on a subset of tumors from the discovery cohort to confirm the mutations found in *RAS*-positive samples. The PCR reaction included 1 μL of cDNA or 50 ng of DNA, 0.2 μM of each specific primer, 1.5-mM MgCl_2_, 0.8-mM dNTP mix and 2 units of Platinum Taq DNA polymerase (Invitrogen) in a 25-μL final volume. The amplified products were analyzed by gel electrophoresis on a 2.0% agarose gel, visualized in the Gel Doc EZ system (Bio-Rad, Hercules, CA, USA) and purified using illustra ExoProStar S (GE Healthcare, Waukesha, WI, USA). Sequencing was performed with the Big Dye TerminatorCycle v3.1 Sequencing Ready Reaction kit in the ABI 3130 platform, according to the manufacturer’s instructions (Applied Biosystems, Foster City, CA, USA). All samples were sequenced at least twice.

### 2.7. In Silico Analysis 

The HOPE server (https://www3.cmbi.umcn.nl/hope/, accessed on 28 September 2020) was used to predict the structural and functional effects of a point mutation on protein 3D structure and function. For this, server relies on sequence annotations from the UniProt database, predictions from the Reprof software for a mutational analysis and calculations on the 3D protein structure [36]. 

### 2.8. Differentially Expressed Genes Analysis in a RAS-Positive Cohort

R package DESeq2 (version 1.30.0) [37] was used to analyze differentially expressed genes (DEGs) amongst *RAS*-positive samples and 16-gene mutation panel negative samples. The adjusted *p*-value ≤ 0.05 was taken as the cut-off value (Figure 1). A total of 1765 genes were found with significantly different expressions in the discovery cohort, 1280 downregulated and 485 upregulated. For biological function and pathway enrichment, the top 10% differentially expressed genes were filtered, resulting in a list of 177 genes with the lowest adjusted *p*-values. 

### 2.9. TCGA Cohort Mutation Analysis 

The RNA-seq data of 492 PTC and 58 normal thyroid samples from the TCGA cohort were downloaded from the TCGA portal (https://portal.gdc.cancer.gov, accessed on 24 July 2020). To detect somatic variants, the maf open file created with Mutect was used. In this file with annotation, *RAS*-positive PTC samples (*n* = 60) were compared to the remaining PTC samples, called group “others” (*n* = 432), including those with BRAF mutations. Maftools was used to detect recurrent mutations exclusively found in the *RAS*-positive group. The list of exclusive genes was enriched to signaling pathways using KEGG data through Enrichr (https://maayanlab.cloud/Enrichr/, accessed on 12 July 2020).

### 2.10. TCGA Expression Analysis

R package DESeq2 was used to identify DEGs between 60 PTC harboring *RAS* mutations versus 550 *RAS*-negative samples, hereafter named the others group, including normal adjacent samples. The adjusted *p*-value < 0.05 was taken as the cut-off value. Hence, the top-ranked differentially expressed genes (top 10%) were submitted to a gene set enrichment analysis (GSEA) and Kyoto Encyclopedia of Genes and Genome (KEGG) enrichment analysis, as described for the discovery cohort in Figure 1.

### 2.11. Pathway Enrichment Analysis Using GSEA and KEGG 

To mine significant biological processes and pathways we used as input the list of recurrent and exclusively mutated genes and 10% DEG in both the discovery cohort and TCGA cohort analysis. Gene set enrichment analysis (GSEA), which focused on the annotation of gene sets to the biological process, molecular functions and cellular components, was performed through the gseGO function (default parameters: nPerm = 10,000, minGSSize = 3, maxGSSize = 800, *p* = 0.05) [38,39].

ClusterProfiler, an R/Bioconductor package (version 3.16.1), and its R function gseKEGG (nPerm = 10,000, minGSSize = 3, maxGSSize = 800, *p* = 0.05; if any pathway was enriched under this *p*-value, more ample values were used, since the gene-enriched list holds the ones with the lowest adjusted *p*-values) were used for Kyoto Encyclopedia of Genes and Genome (KEGG) pathway enrichment [40]. Further, gageData and pathview packages were used to evaluate specific signaling pathways, once this package draws KEGG pathway maps shading the molecules according to their degree of up- and downregulation [41]. 

### 2.12. Expression Analysis by Quantitative RT-PCR (qRT-PCR)

To validate the signaling effect triggered by the presence of mutations, we measured the mRNA expression of downstream target genes by qRT-PCR. Total RNA (1 μg) was treated with DNAse and reverse-transcribed into cDNA with 50-μM oligo(dT)20 using a Superscript III transcriptase kit (Thermo Fisher Scientific). About 2 μL of cDNA was used in a 12-μL PCR reaction containing 1× SYBR Green PCR Master Mix (Applied Biosystems) and 3 pmol of each specific primer for the target gene. As a reference gene, we used ribosomal protein S8 (RPS8). The reaction was performed in triplicate on a QuantStudioTM 12K Flex (Applied Biosystems). The relative expression levels were calculated based on Delta-Delta-CT (ddCT), as previously reported [42,43]. The Kruskal-Wallis test was applied to analyze our set of candidates.

## 3. Results

### 3.1. Outline of RNA-Seq Data

We generated RNA-Seq data for 11 *RAS*-mutated thyroid cases with 89 million total reads per sample on average (35–163 million reads) (Table 1). About 99% of the reads were mapped to the human reference genome. The percentage of reads with Quality Q20, a sequencing score quality that indicates a call accuracy of 99%, ranged from 89.6% to 93.2%.

### 3.2. Somatic Single-Nucleotide Variants in the RAS-Positive Cohort

To detect Somatic Single-Nucleotide Variants (SNVs) that are likely clinically relevant and cooperate with *RAS* in the pathogenesis or progression of thyroid cancer, we extensively explored the RNA-Seq data by using two major approaches: (i) by calling SNVs and Indels and (ii) by exploring the gene expression profile (see pipeline detailed in Figure 1). The SNVs were filtered to keep only rare variants (population allele frequency <0.1%) using the gnomAD and the largest online archive of Brazilian mutations (ABraOM), with a total of 2,382,573 variants [34]. Data from Encode [44] was also used to remove variants present in normal thyroid tissue.

**Figure 2:**
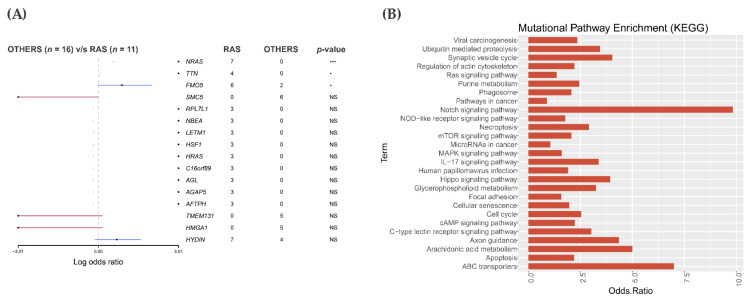
Mutational analysis and pathway enrichment in the *RAS*-positive cohort. (**A**) Forest plot shows representative genes that were recurrently mutated in the 11 *RAS*-positive groups or in the “others” group (samples negative for the driver mutation). The black points indicate genes exclusively mutated in the *RAS* cohort. The blue line shows genes mutated in both groups and the red line the ones mutated exclusively in the “others” group. Recurrent mutations were considered those mutations that occurred in at least two samples. *** *p*-value = 0.001, * *p*-value ≤ 0.05, NS = non-significant *p*-value (**B**) A KEGG pathway enrichment of the 126 exclusively mutated in the *RAS* cohort.

After a comparison of *RAS*-positive samples with 16 panel negative samples, we obtained a list of 126 genes that were exclusively and recurrently altered in the *RAS*-positive cohort (Figure 2A and Figure S1). Those genes were enriched for pathways using the KEGG database through Enrich.

### 3.3. Pathway Enrichment Analysis in the RAS-Positive Cohort 

Numerous pathway databases have been made available to infer biologically relevant pathway activity. To gain insights into pathways affected by *RAS*-associated mutations, the list of all 126 genes exclusively and recurrently mutated in this cohort was analyzed with the Enrichr web-based application (http://amp.pharm.mssm.edu/Enrichr, accessed on 28 July 2020), using the KEGG pathway information [45,46]. The data was plotted with odds ratio values calculated based on the total number of genes in each pathway and how many of them were found mutated (Figure 2B).

A pathway was considered affected if it contained at least two or more mutated genes. We found between two and five genes mutated in the same pathways. Some genes were found to be affecting multiple pathways. A plot with the number of genes affected (odds ratio) in each pathway group is shown (Figure 2B). The pathways that present higher odds ratios and *p*-values < 0.05 are: Axon guidance (5/181 genes: *ABLIM1*, *PAK1*, *UNC5B*, *SEMA3D* and *RHOD*); Hippo (4/160: *PAK1, RASSF4, HIPK2* and *DVL1*); Notch (3/48 genes; *HDAC2, DVL1* and *PSEN2*) and ABC transporters (2/45 genes; *ABCC4* and *ABCA5*). Among them, the Hippo pathway has been recurrently associated with tumor progression and selected for further validation. 

Sanger sequencing confirmed the presence of the mutations of *PAK1, RASSF4*, *HIPK2* and *DVL1* in the discovery cohort (Figure S2A,B). 

### 3.4. In Silico Analysis of Hippo Pathway Mutated Genes in RAS-Positive Samples and Its Mutational Impact

**Figure 3:**
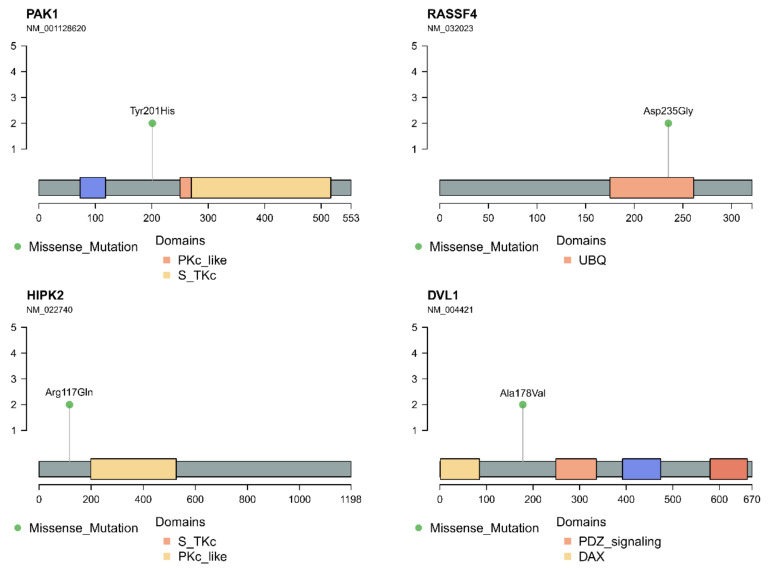
Amino acid change and localization relative to the respective protein domains in the four proteins (PAK1, RASSF4, HIPK2 and DVL1) whose corresponding genes belong to the Hippo pathway and are mutated in a *RAS*-positive cohort. Brackets from 1 to 5 indicate the number of samples positive for each mutation. In our *RAS*-positive samples, the alteration of each gene was present in two samples.

The *PAK1* missense mutation (rs775172015) promoted a tyrosine to a histidine substitution at codon 201 (p.Y201H) (Figure 3). Tyrosines 153, 201 and 285 were reported as required for a PAK1 kinase activity and identified as a JAK2 tyrosyl phosphorylation site by mass spectrometry and two-dimensional peptide mapping [47]. In fact, this alteration was classified as deleterious (sift score 0.04) and possibly damaging (PolyPhen2 score 0.789). The HOPE analysis indicated that the p.Y201H substitution was located within a stretch of residues annotated in UniProt as a special region of interaction with CRIPaK, a cysteine-rich inhibitor of Pak1 (CRIPak). Therefore, the difference in amino acid properties between the wild type and the mutant likely affects its function. Due to the smaller size and less hydrophobicity of the mutant residue, hydrophobic and other interactions either in the core of the protein or on the surface may be lost.

The *RASSF4* missense mutation resulted in the p.D235G change and was classified as tolerated (sift score 0.25) and benign (PolyPhen2 score 0.075) (Figure 3). Interestingly, the mutation is located within the Ras associating site, which interacts with the Ras GTPase protein family, controlling cellular processes such as membrane trafficking, apoptosis and proliferation. The changed amino acid found in RASSF4 is smaller, neutral and more hydrophobic than the wild type (negative charge), which can lead to a loss of interaction and, also, a loss of hydrogen bonds and/or disturb the correct folding. The mutation introduced a glycine, a very flexible amino acid, likely disturbs the required rigidity of the protein at this position.

The *HIPK2* mutation promoted the p.R117Q substitution, previously described in the dbSNP database (rs764542823) and classified as tolerated (sift score 0.2) and probably damaging (PolyPhen2 score 0.978) (Figure 3). The HOPE analysis revealed that the mutant and the wild-type amino acid differ in size and charge. While the wild type is positive, the mutant is neutral and smaller. The p.R117Q mutation is located within a stretch of residues annotated in UniProt as a transcriptional co-repression site. As the wild-type residue is highly conserved, the effect of replacing the R117 of HIPK2 with other residue that has differences in amino acid properties likely disrupt the three-dimensional structure of the protein and its function. Although neither the mutant residue described here nor other residue with similar properties was described at this site, based on conservation scores the mutation is probably damaging. 

The DVL1 mutation promoted the p.A178V substitution previously described in the dbSNP (rs139645212) as deleterious (sift score 0.02) and benign (PolyPhen2 score 0.036) (Figure 3). The interaction between the DLV protein and YAP/TAZ rely on specific conserved domains of the protein, like N-terminal DIX, central PDZ and c-terminal DEP domains. Studies indicate that the PDZ domain and WW domain, present in both the YAP and TAZ resulting proteins, are responsible for their interaction. The change described in this study is located next to a PDZ domain, which may interfere with this interaction.

Additionally, the integrative Onco Genomics analysis (intOgen https://www.intogen.org/search, accessed on 2 December 2020), which evaluated the mutational status in 28,076 samples of 66 cancer types, acknowledged different mutations in *PAK1*, *RASSF4*, *DVL1* and *HIPK2* genes. Recurrent mutations in these genes in many types of cancer suggest their action in the tumorigenesis process (Figure S3A) [48]. 

### 3.5. Mutational Landscape and Pathway Enrichment in the RAS-Positive Samples from TCGA Cohort

**Figure 4:**
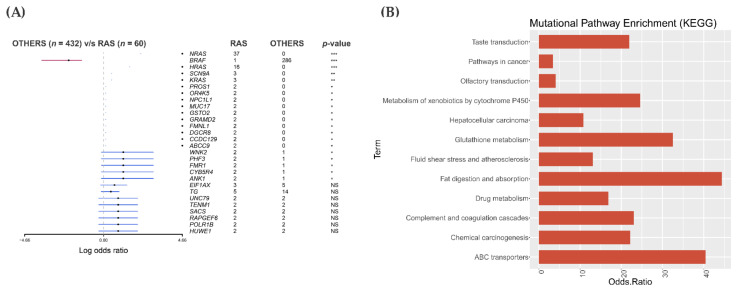
Mutational analysis and pathway enrichment (KEGG) in *RAS*-positive papillary thyroid carcinomas (PTC) from The Cancer Genome Atlas (TCGA) cohort. (**A**) A forest plot showing the eleven genes exclusively and recurrently mutated (present in at least two samples) in 60 RAS-positive samples. *** *p*-value ≤ 0.001, ** *p*-value ≤ 0.01, * *p*-value < 0.05, NS = non-significant *p*-value (**B**) KEGG pathway enrichment of the 11 exclusively mutated genes in rhe RAS-positive TCGA cohort.

The analysis of 60 *RAS*-positive PTCs from the TCGA cohort was performed against the 432 PTCs designated as “others”. As expected, *NRAS*, *HRAS* and *KRAS* showed the highest mutation rank in the *RAS*-mutated group. Other 11 genes were mutated recurrently and exclusively in the *RAS*-positive PTC samples (Figure 4A). BRAF V600E was the most frequently mutated gene in the PTC group classified as others. None of the genes that were mutated in our *RAS*-positive samples were detected in the *RAS*-positive samples from the TCGA cohort, including *PAK1, RASSF4, HIPK2* and *DVL1*, (Figure S3B). However, most of our *RAS*-positive samples are FTC and FVPTC while the *RAS*-positive PTC samples from TCGA cohort are the classic variant of PTC.

The pathway enrichment analysis showed that most of the 11 genes belonged to one pathway. Some of the genes were enriched for more than one signaling pathway. The odds ratio varied according to the number of genes mutated in each pathway (Figure 4B). 

Remarkably, only two pathways that were enriched in the *RAS*-positive PTC from the TCGA cohort were also found in our *RAS*-positive cohort: ABC transporters (*p*-values ≤ 0.05) and pathways in cancer. However, none of the genes (*ABCC9* and *GSTO2*) mutated in the TCGA were found mutated in our set of samples.

### 3.6. Gene Expression Pattern in the RAS-Positive Samples

**Figure 5:**
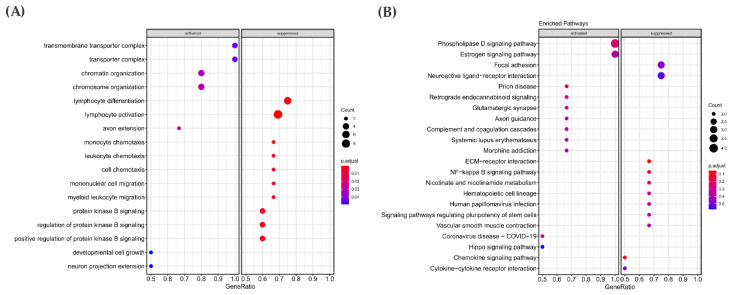
Differential gene expression analysis in the *RAS*-positive group from the discovery cohort (11 samples) detected 1765 genes significantly up- and downregulated. The top 10% ranked genes were differentially expressed, which shows that the ones with the lowest adjusted *p*-values were submitted to GSEA and KEGG enrichment to gain insight into altered biological processes and pathways. (**A**) GSEA enrichment analysis of biological processes that contain upregulated genes are marked on the left panel and downregulated on the right panel. (**B**) Pathway enrichment analysis (KEGG) results showed pathways in which most of their genes are upregulated (left) or down-regulated (right) in the *RAS*-positive cohort.

By comparing *RAS*-positive samples from the discovery cohort against the “others”, we identified 1765 genes that were found differentially expressed (adjusted *p*-value ≤ 0.05). About 485 genes were upregulated in the *RAS*-positive samples, while 1280 genes were downregulated. To gain insights into altered pathways, we performed the Gene Set Enrichment Analysis (GSEA and KEGG), using top 10% ranked genes DEGs (177 genes up- and down-regulated with the lowest adjusted *p*-value) (Figure 5).

GSEA functional analyses revealed that genes involved in cell growth development, chromatin organization and chromosome organization were differentially expressed (Figure 5A). KEGG pathway enrichment showed that pathways such as NF-Kappa beta and ECM receptor interaction contained genes that were downregulated in the discovery cohort. Among the pathways whose genes were enriched as upregulated, the most interesting was the Hippo signaling pathway (Figure 5B). Remarkably, this pathway showed significant enrichment in the discovery cohort in both mutational and gene expression analysis.

Next, to verify if the 11 *RAS*-positive samples in the discovery cohort and the TCGA *RAS*-positive samples show similar expression profile, the BAM files from 60 *RAS*-positive samples and 25 BRAF V600E-positive PTC samples from TCGA were processed according to our pipeline (Figure 1), removing the batch effect due to the difference in the library construction. A principal component analysis (PCA) performed on DEGs showed that the *RAS*-positive samples from a discovery cohort showed an expression profile similar to that observed in *RAS*-positive samples from the TCGA cohort, as most samples grouped into the same cluster. Instead, the BRAF V600E-positive samples from the TCGA cohort grouped into a distinct cluster (Figure S4).

Accordingly, an enrichment analysis was also performed in the 10% ranked genes obtained after performing differential gene expression (adjusted *p*-value ≤ 0.05) in the 60 *RAS*-positive PTC from the TCGA cohort (1700 genes with the lowest adjusted *p*-value).

**Figure 6:**
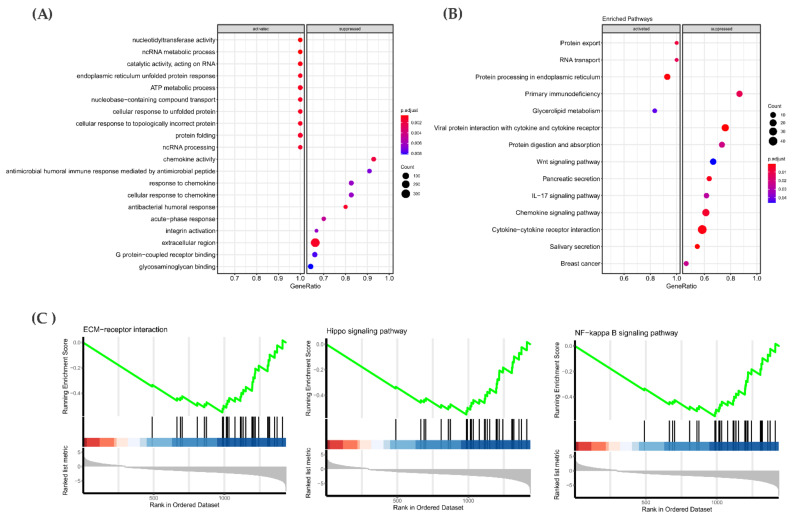
Differential gene expression analysis in *RAS*-positive PTC samples from the TCGA cohort (60 samples). Enrichment analysis (GSEA and KEGG) was performed on the top 10% genes differentially expressed composing the ones with lowest adjusted *p*-values to gain insights into altered biological process and pathways. (**A**) GSEA enrichment analysis of the biological processes that contain upregulated genes are marked on the left panel and downregulated on the right panel. (**B**) Pathway enrichment analysis result showed pathways in which most of their genes are upregulated (left) or downregulated (right) in *RAS*-positive TCGA cohorts. (**C**) Common pathways associated with cancer progression differentially expressed in both *RAS*-positive discovery cohort and *RAS*-positive PTC from the TCGA cohort.

The GSEA functional analyses showed that genes involved with nucleoside and ATP metabolism were upregulated, and the genes associated with cellular processes such as the regulation of DNA replication were downregulated (Figure 6A). Regarding KEGG pathway enrichment, the pathway with the larger number of genes differentially expressed refers to the immune response (Figure 6B).

Among the pathways already known to contribute to cancer progression, three of them were enriched in both a *RAS*-positive discovery cohort (11 samples) and *RAS*-positive PTC from a TCGA cohort: ECM-receptor interaction, NF-kappa B and Hippo signaling (Figure 6C).

**Figure 7:**
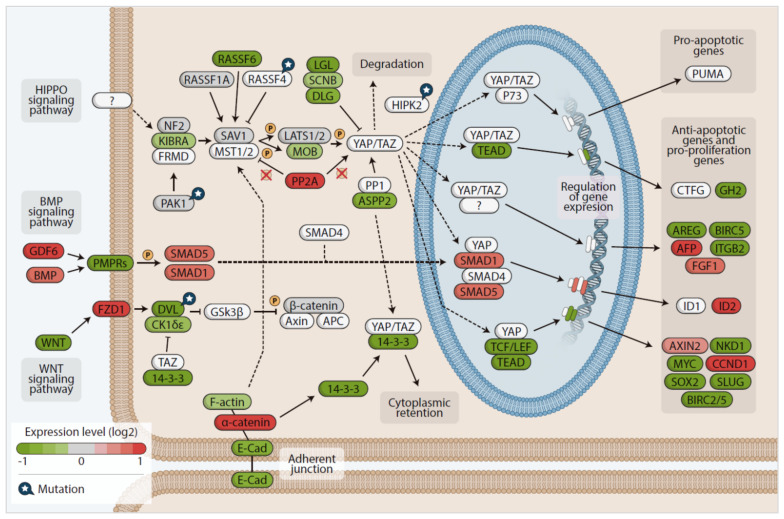
Hippo signaling pathway KEGG-based map, showing genes up- (red) and downregulated (green) detected by differential expression (adjusted *p*-value ≤ 0.05) performed in both the *RAS*-positive discovery cohort and *RAS*-positive TCGA cohort.

We used the gageDate package to analyze which genes were altered in the Hippo pathway in *RAS*-positive samples from both discovery and the TCGA cohorts. The data are summarized in Figure 7. Briefly, the genes *RASSF6*, *DLG1*, *AREG*, *ITGB2*, *BIRC2*, *YWHAQ* (14-3-3), *BMPR1B* and *WNT11* were downregulated, while the genes *GDF6*, *ID2*, *CCND1* and *FZD1* were upregulated. Remarkable, *GDF6* was one of the most upregulated, with an expression 6.97-fold higher than its expression in the others group. The fact that *GDF6* and *FZD1* are upregulated may indicate a crosstalk between BMP, Wnt and Hippo signaling, which is reinforced by the fact that the target genes of both pathways are highly expressed (*ID2* and *CCND1*, respectively).

Another interesting result assembling our mutational and differential expression analysis is the fact that the *PAK1* gene found exclusively mutated in the *RAS*-positive discovery cohort was also found significantly upregulated (0.5-fold) (*p*-value= 0.002, adjusted *p*-value = 0.043). 

Regarding the *RAS*-positive samples from the TCGA cohort, all the genes above mentioned as downregulated in the *RAS*-positive discovery group were also downregulated in the *RAS*-positive samples from the TCGA cohort. We additionally found other genes that play a significant role-regulating the Hippo pathway downregulated, such as *KIBRA*, *MOB1A* and *LATS1* (Hippo kinase activator) (Figure 7), whereas the *GDF6, ID2* and *SMAD5* from the BMP pathway were upregulated. 

The higher *PAK1* expression, which was concomitant with a decreased *NF2* and *LATS1* expression, strongly suggests that the Hippo pathway kinase core is off, and YAP/TAZ proteins are active in the nucleus. In fact, target genes that are normally expressed when YAP is localized in the nucleus, such as *AFP* and *FGF1*, were upregulated. Finally, another evidence comes from the increased expression of the *PP2R2C* gene, which encodes the protein phosphatase PPA2A that dephosphorylate MST1/MST2, causing the activation of YAP/TAZ. 

DVL genes (*DVL1*, *2* and *3*) and *DSNK1E,* which encodes the CK1-epsilon kinase, were also found downregulated. As DVL suppresses the YAP/TAZ nuclear abundance and TEAD transcriptional activity, these findings indicate that a DVL protein interaction with YAP/TAZ in the cytoplasm is reduced, resulting in the YAP/TAZ nuclear localization and increased TEAD activity. A possible activation of the Wnt pathway is reinforced by the fact that *AXIN2* and *CCND1,* target genes of this pathway that are normally expressed when YAP is in the nucleus, showed increased expressions.

Remarkable, the genes encoding proteins that regulate the canonical TGFβ signaling (*TGFBI*, *TGFBR1*, *TGFB2*, *TGFB3*, *TGFBR3*, *SMAD2* and *SMAD3*), showed lower expression, indicating that the TGFβ pathway is likely inactive in the *RAS*-positive samples from the TCGA cohort. 

Altogether, the results of the differential gene expression in the *RAS* data from discovery and the TCGA cohorts suggest that the Hippo pathway is inactive in *RAS*-positive thyroid carcinomas, and, therefore, YAP/TAZ might be localized in the nucleus regulating genes that act in cell proliferation, migration and survival. Moreover, the activation of key genes belonging to the BMP and Wnt pathways raises the possibility of a hypothetical crosstalk among these pathways. 

### 3.7. Gene Expression Analysis: Proof of Concept 

In order to test the hypothesis that the Hippo pathway might be dysregulated and, therefore, YAP/TAZ were able to locate in the nucleus and regulate the genes associated with Wnt and BMP signaling, we performed real-time qPCR of key genes associated with these pathways: *YAP1*, *PAK1*, *NF2*, *LATS1*, *ID1*, *SMAD1*, *SMAD5* and *DVL1*. The expression analysis was performed in five PTC-positive for RAS mutations. As controls, five PTC-positive for BRAF V600E and five normal adjacent thyroid samples were used.

*YAP1* showed a higher expression in *RAS*-positive samples when compared to BRAF V600E-positive PTC (*p* < 0.05). Although not considered significant, BRAF V600E-positive PTCs showed lower levels of *YAP*1 expression than the normal thyroid tissue. *PAK1* also showed a higher expression in the *RAS* group compared to BRAF V600E group (Figure S5). 

On the other hand, the *NF2* expression was significantly diminished in the *RAS* cohort compared to normal adjacent tissue. Remarkable, *NF2* and *LATS1* were also significantly downregulated in the *RAS*-positive PTC from the TCGA cohort (Figure 7). 

Considering the genes belonging to the BMP and Wnt pathways, the *SMAD1* expression was significantly higher in the *RAS* group compared to the BRAF group. *SMAD5* did not show a significant expression difference when compared to both the BRAF and normal adjacent groups. The *ID1* expression was significantly lower in the BRAF group compared to normal tissue. These results, especially *SMAD1* upregulation, are in agreement with TCGA-*RAS* differential expression analysis and reinforce the fact that BMP is active when the Hippo pathway is inactive (Figure S5). 

The difference of LATS1 and DVL1 expression was not considered significant when compared *RAS* group with BRAF V600E and normal thyroid adjacent groups.

## 4. Discussion

In recent years, several studies identified genes that cooperate with *RAS* mutations in the pathogenesis or progression of thyroid and other types of cancers, such as *NF1*, *APC*, *PTEN*, *KEAP1*, *NF2* and others [49,50,51,52,53].

Mathematical prediction predicted synergy between specific combinations of mutations. The subsequent analysis of Pan-Cancer TCGA dataset and Cancer Cell line Encyclopedia (CCLE) uncovered that *NF1* mutations promote cancer not only by increasing Ras signaling but, also, by increasing the number of mutations that would further increase the Ras signaling. In other words, the number of potential noncanonical and canonical *RAS* point mutations capable of promoting cancer was greater in the NF1-deficient context [50].

Others demonstrated that the loss of *Nf2* or Ras activation was insufficient to independently induce thyroid cancers, while the cooperation of Ras and Nf2 led to poorly differentiated thyroid carcinomas with increased MAPK signaling [53]. In human thyroid cancers, a high frequency of 22q loss was preferentially associated with *RAS*-mutated PTC and poorly differentiated thyroid carcinomas as compared to BRAF-mutated tumors. Remarkably, the authors demonstrated that Nf2 loss promoted Ras signaling, in part through Hippo pathway inactivation and YAP-induced transcriptional activation of oncogenic and wild-type *RAS* [53].

The Hippo pathway regulates tissue growth and cell fate. It was first postulated to play a role in human cancer on the basis of the notorious overgrowth of *Drosophila melanogaster* tissues that harbor mutations in genes that encode proteins associated with this pathway [54,55,56].

The core of the Hippo pathway consists of the serine/threonine kinases MST1/2 and LATS1/2. MST kinase activates LATS, via a membrane-associated complex containing the tumor suppressor NF2, which, in turn, phosphorylates the transcriptional coactivators YAP and TAZ on multiple sites. Once phosphorylated, the YAP/TAZ complex is inactivated through cytoplasm sequestration via binding to 14-3-3 or by its increased ubiquitination and degradation. Conversely, inhibition of the Hippo pathway leads to dephosphorylation of the YAP/TAZ complex, its increased nuclear abundance and transcriptional enhancer activation domain (TEAD) transcriptional activity, which promotes cell proliferation [56,57]. Beside the TEAD family of transcription factors, YAP/TAZ also interacts with other transcription factors, including Smad, p63 and PAX [56,58,59].

Although mutations and altered expressions of a group of Hippo pathway genes that lead to the increased activity of coactivators YAP and TAZ and an overgrowth phenotype have been observed in human cancer [56,60,61], the core Hippo pathway genes are infrequently mutated. As far as we know, *NF2* is the only commonly mutated driver gene in the Hippo pathway in thyroid cancer [56].

Importantly, beyond the main components of the Hippo pathway, several upstream regulatory branches have been reported to modulate the Hippo pathway, such as Wnt and BMP/Transforming Growth Factor β [59].

To identify genes that cooperate with *RAS* in thyroid tumorigenesis, in this study, we performed an RNA-Seq analysis of FVPTC and FTC with the canonical *RAS* mutation. Here, we describe missense mutations in four components of the Hippo pathway. 

**Figure 8:**
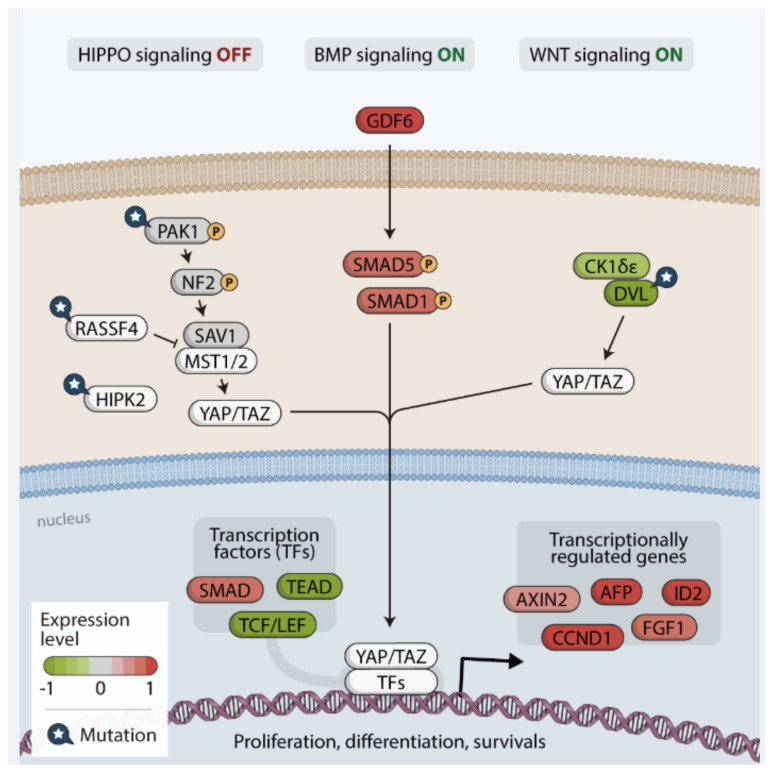
Hippo pathway simplified map, summarizing the findings of the mutation and differential gene expression analysis in our *RAS*-positive cohort supported by the *RAS*-positive TCGA data analysis. Inactivation of this pathway promotes YAP/TAZ translocation into the nucleus and targets gene expression, which leads to cellular survival and growth. Once Hippo is inactive and YAP/TAZ acting, a crosstalk may occur with the BMP and Wnt pathways.

The genes *PAK1* (p21-activated kinase 1), *RASSF4* (RAS association domain-containing family protein), *HIPK2* (homeodomain-interacting protein kinase 2) and *DVL1* (the human homolog of the Drosophila dishevelled gene) were mutated in the FVPTC and FTC samples (Figure 8). 

The mutations found in the *PAK1* and *HIPK2* genes may affect the Hippo pathway functions through *NF2*-reduced expression and YAP/TAZ phosphorylation, respectively. Although controversial, it has been suggested that these proteins promote Hippo pathway repression, YAP/TAZ increased nuclear abundance and the transcriptional activity of several transcription factors, including TEAD [62,63,64,65,66,67]. 

Though further studies are needed to clarify whether HIPK2 regulates YAP phosphorylation and its activity either in the cytoplasm or at a nuclear level, the mutation described here likely interferes with the HIPK2 function and transcription regulation of Hippo target genes [67,68]. 

Interestingly, HIPK family members are reported to have distinct and contradictory effects on cell proliferation and tissue growth [69,70]. The *HIPK2* gene is located on chromosome 7q, near the *BRAF* locus. The increased expression of *HIPK2* was reported in sporadic pilocytic astrocytoma, likely due to the copy number gain [71]. *HIPK2* amplification was also reported in melanoma [72]. Moreover, it has been reported that HIPK2 promotes the activation of conserved pathways implicated in cancer, such as Wnt and Hippo [73].

It is known that the Hippo and Wnt pathways equally regulate the nuclear transcriptional activity and are closely connected to each other on multiple levels [74,75,76]. It has been reported that CK1ɛ isoform phosphorylates YAP/TAZ after priming phosphorylation from LATS1/2 and following CK1ɛ binding to MST1/2. Under these conditions phosphorylation of DVL is reduced, leading to inhibition of Wnt Signaling. Also, YAP/TAZ complex bind to the cytoplasmic DVL1, preventing phosphorylation and abrogating the complex translocation to the nucleus [74,75]. The A178V DVL1 substitution here described may promote Wnt activation and the expression of target genes by interfering with YAP/TAZ binding sites or preventing the CK1-dependend phosphorylation of DVL1.

*RASSF4*, a member of tumor suppressor genes family, is broadly expressed in normal tissues. However, it was found down regulated in several tumors subtypes and cancer cell lines by promoter methylation. RASSF proteins can associate, via its SARAH domain, with downstream kinases of Hippo pathway such as MST1/2 and SAV1 in order to promote apoptosis. Therefore, mutations describe here may affect its ability to associate with these kinases and Hippo pathway signalling [62]. 

While Hippo genes have been rarely reported as mutated in human cancers, the recurrent mutations reported in *PAK1*, *RASSF4*, *HIPK2* and *DVL1* in many types of cancer by the Integrative Onco Genomics, a web site that explores driver genes in cancer, suggest they likely play a role in tumorigenesis process. 

Beyond the main components of the Hippo pathway described above as mutated, many other additional proteins that have been reported to modulate this pathway were dysregulated at expression levels. For example, *RASSF6, KIBRA*, *NF2*, *LATS1* and *MOB1A* showed lower expression in *RAS*-positive samples from both discovery and TCGA cohorts, suggesting YAP/TAZ reduced phosphorylation in *RAS* positive samples.

We additionally found increased expression of *PAK1* in *RAS* positive samples. Hence, *PAK1* increased expression, associated with *NF2* and *LATS1* reduced expression, indicates a new potential regulation mechanism of Hippo pathway inactivation in *RAS*-positive samples.

Reduced expression of tumor suppressor NF2, due to 22q loss, was previously associated with *RAS* mutant poorly differentiated thyroid carcinoma, undifferentiated thyroid carcinoma and in PTC from TCGA cohort [53]. The authors demonstrated that loss of *Nf2* or *Ras* activation is insufficient to independently induce thyroid cancer in mice, but their combination was highly tumorigenic. They suggested that *NF2* loss cooperates with mutant *RAS* to increase signaling via MAPK, acting in part through YAP-induced transcriptional activation of oncogenic and wild-type *RAS*, providing a novel mechanism of promotion of *RAS*-induced [53].

As Hippo pathway suppresses cell growth through phosphorylation of YAP, which disrupts its ability to promote TEAD-dependent transcription of genes involved in proliferation and survival [77], we next investigated the expression of YAP/TAZ target genes. 

We here observed an increased expression of YAP/TAZ target genes *AFP*, *FGF1*, suggesting that YAP/TAZ complex was able to promote their transcriptional activation. Another relevant finding was that *ID2*, *CCND1*, and *AXIN2* genes, associated with BMP and Wnt pathways, showed increased expression. These findings raised the hypothesis of a crosstalk between Hippo, BMP and Wnt signalling pathways. In other words, nuclear YAP might be able to bind to other transcriptions factors, other than TEAD, to activate transcriptional program.

The TCGA cohort analysis corroborates with this hypothesis since all three DVL genes were down regulated and the *AXIN2*, *CCND1*, *CTNNB1, AXIN1* were detected with higher expression in this cohort, suggesting that Wnt signalling pathway was active. In addition to the Wnt target gene *CCND1*, we found increased expression of *FZD1* (in both cohorts), which triggers Wnt activation.

Another finding that corroborates with our hypothesis is the fact that we found increased expression of *GDF6*, also named *BMP13*, in the *RAS*-positive discovery cohort reported in this study and in PTC from TCGA cohort. GDF6 is a BMP ligand that was previously described as an oncogene in melanoma. Its increased expression was detected in melanoma, compared to the normal tissue and benign skin lesions [78]. 

Furthermore, increased expression of other components of BMP signalling pathway, *SMAD5* and *SMAD1*, were identified in *RAS*-positive samples from the TCGA cohort. These findings, in association with further validation analysis of SMAD1 in our *RAS* cohort, reinforce the possibility that activation of the BMP pathway leads to YAP/TAZ shift to the nucleus to induce target gene expression.

Altogether, our findings suggest that the Hippo pathway is dysregulated in thyroid cancer *RAS*-positive samples, resulting in the translocation of the YAP/TAZ complex to the nucleus. Hence, the pathway inactivation can occur not only through *NF2* loss but also through mutations or changes in the expression of genes that act or regulate proteins that act in the kinase core. Once in the nucleus, YAP/TAZ complex can interact with sequence specific transcription factors other than TEADs. The complex can cooperate with SMADs and T-cell specific transcription factor (LEF/TCF), among others, to regulate expression of Hippo target genes such as *AFP*, *FGF1*, *ID2*, *AXIN2* and *CCND1* that are involved in cell proliferation and cell survival. Besides, BMP and Wnt pathways are active due to a cross talk with effectors of Hippo signalling (Figure 8). Our data suggest a possible mechanism whereby dysregulation of the Hippo pathway and activation of pathways such as BMP and Wnt may contribute for tumorigenesis in *RAS*-positive tumors. If the mechanism of Hippo dysregulation described here hyperactivate MAPK signalling needs further investigation.

As it has been shown that this network contributes to thyroid cancer progression, we believe that these findings will not only help diagnosis and prognosis of thyroid cancer but also the identification of new therapeutic approaches for targeting the whole Hippo pathway, therefore, helping to improve the treatment of thyroid cancer. 

## 5. Conclusions

In summary, our study suggests that mutations and/or genes expression alteration that lead to Hippo pathway inactivation can contribute to tumorigenesis in *RAS* positive thyroid tumors. The mutations found in *PAK1*, *HIPK2* and *RASSF4* genes may affect Hippo pathway function through *NF2* reduced expression, YAP/TAZ phosphorylation, and reduce association with MST1/2 and SAV1 respectively. Also, *DVL1* substitution may promote Wnt activation and the expression of target genes by interfering with YAP/TAZ binding sites or preventing the CK1-dependend phosphorylation of DVL1.

Increased expression of YAP/TAZ target genes *AFP*, *FGF1* were observed, suggesting that YAP/TAZ complex is likely in the nucleus promoting their transcriptional activity, an event only possible if Hippo pathway is inactive. *RASSF6*, *KIBRA*, *NF2*, *LATS1*, *MOB1A*, down regulation corroborates with these findings. On the other hand *AXIN2*, *CCND1*, *CTNNB1, AXIN1, FZD1* higher expression in this cohort, suggest Wnt activity. *GDF6*, *ID2*, *SMAD5*, and *SMAD1* up-regulation reinforce the hypothesis of activation of the BMP pathway. 

In summary, our findings suggest that the Hippo pathway is dysregulated in thyroid cancer, mainly in *RAS*-positive tumours. Our data also suggests a cross talk of Hippo with Wnt and BMP pathways, providing potential mechanistic bases for the synergism between RAS, Hippo, Wnt and BMP pathways and, thus, novel opportunities for effective targeted therapies. 

## Figures and Tables

**Table 1:** Clinical pathological features of the *RAS*-positive cohort and sequencing coverage.

Sample	*RAS* Mutation	No. of Reads (Millions)	Reads Mapping Mutant Allele (%)	Tumor Type *	Tumor Size (cm)	Multifocal	Extrathyroidal Extension	Vascular Invasion	Metastases	Recurrence	Age at Diagnosis	Gender
1	N Q61R	136	66	FVPTC	5.0	N	Y	N	N	N	32	M
2	N Q61K	113	62	FVPTC	4.0	N	N	N	N	N	40	M
3	N Q61R	75	78	FVPTC	5.0	Y	N	N	N	N	28	F
4	N Q61R	106	33	FVPTC	1.7	Y	N	N	N	N	55	F
5	N Q61R	118	56	FVPTC	3.0	N	N	N	N	N	36	F
6	H Q61R	242	71	FVPTC	1.1	N	N	N	N	N	45	F
7	N Q61R	93	70	FTC	3.5	Y	Y	N	Y	N	45	F
8	K Q61R	168	22	FTC	3.2	N	N	Y	N	N	48	F
9	N Q61R	131	50	FTC	1.6	N	Y	Y	N	N	48	F
10	H Q61K	91	59	FTC	7.5	N	Y	Y	Y	N	76	F
11	H A11G	117	13	FTC	10.0	N	Y	Y	Y	Y	70	M

* No anaplastic or dedifferentiated components were described in these samples. FVPTC, follicular variant of papillary thyroid carcinoma, FTC, follicular thyroid carcinoma, Y = yes and N = no; F: female; and M: male.

## Data Availability

TCGA data used in this project can be access at https://portal.gdc.cancer.gov, accessed on 24 July 2020, ENCODE normal thyroid can be found at https://www.encodeproject.org, accessed on 20 February 2019.

## Supplementary Materials

The following are available online at https://www.mdpi.com/article/10.3390/cancers13102306/s1, Figure S1: List of 126 genes found exclusively and recurrently mutated in *RAS* discovery cohort (11 samples). The 11 *RAS*-mutated samples were classified according tumor histology (FTC and FVPTC), stage and risk for recurrence. Figure S2: (A) Distribution of *PAK1*, *RASSF4*, *HIPK2*, and *DVL1* in *RAS*-positive discovery cohort, the four genes are mutated exclusively in the *RAS* group. (B) Sanger sequencing confirming the nucleotide change in the four genes mutated in the Hippo Path: PAK1 (Chr11:77066884, A/G, c.T601C:p.Y201H), RASSF4(Chr10:45486414, A/G, c.A704G:p.D235G), HIPK2 (Chr7:139416484, C/T, c.G350A:p.R117Q) and DVL1(Chr1:1277119, G/A, C533T:p.A178V), Figure S3: (A) *PAK1*, *RASSF4*, *HIPK2* and *DVL1*, mutational distribution in Integrative Onco Genomics cancer cohorts. (B) Mutations found in *PAK1*, *RASSF4*, *HIPK2* and *DVL1* are absent in TCGA-THCA cohort, showing no co-occurrence of mutations in these genes with mutations in the *BRAF* and *RAS* genes, Figure S4: Principal component analysis (PCA) showing a similar expression profile of the *RAS* discovery cohort (11 samples) when compared to *RAS*-positive TCGA-THCA, Figure S5: Real time polymerase chain reaction (qRT-PCR) performed on additional *RAS*, *BRAF* V600E and normal adjacent thyroid tissue (referred as normal adj in graph). Key genes from Hippo, BMP and Wnt signaling pathways altered in *RAS*-positive TCGA-THCA cohort were evaluated. *YAP1*, *NF2* and *PAK1* from Hippo pathway and *SMAD1* and *ID1* from BMP signaling had significant results.
