# Supplementary figures and images for: Evidence of Cooperation between Hippo Pathway and RAS Mutation in Thyroid Carcinomas
Source: Cancers (Basel). 2021 May 12;13(10):2306. doi: 10.3390/cancers13102306 (PMC8151534; doi:10.3390/cancers13102306)

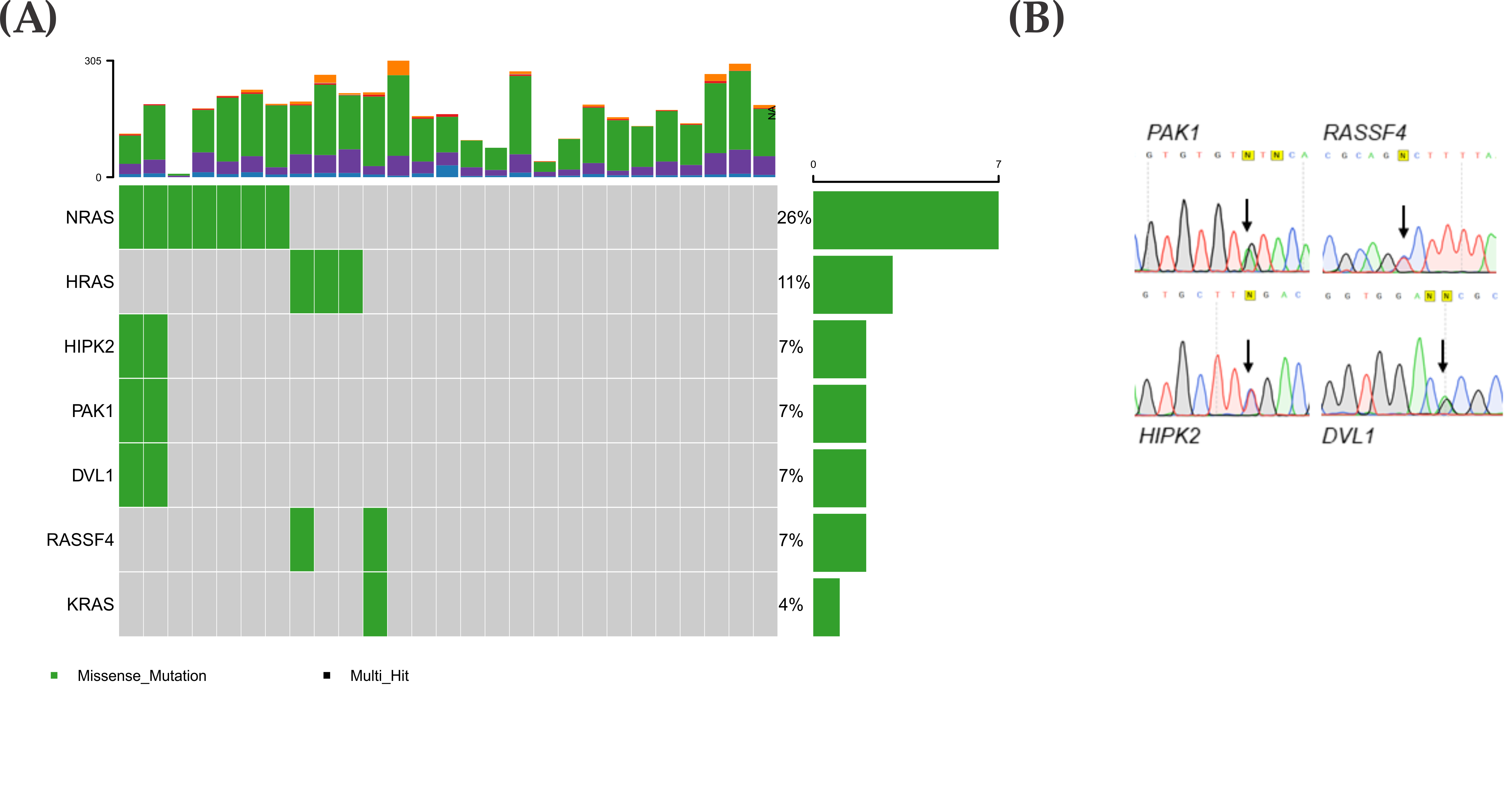

Supplement: Supplementary file 1 [file cancers-13-02306-s001.zip › Figure S2.tif]
